# Catalpol protects mice against Lipopolysaccharide/D-galactosamine-induced acute liver injury through inhibiting inflammatory and oxidative response

**DOI:** 10.18632/oncotarget.23242

**Published:** 2017-12-12

**Authors:** Haogang Zhang, Ruichun Jia, Fujing Wang, Gongcai Qiu, Pengfei Qiao, Xunzheng Xu, Dequan Wu

**Affiliations:** ^1^ Department of General Surgery, The Second Affiliated Hospital of Harbin Medical University, Harbin, Heilongjiang 150086, China; ^2^ Department of Blood Transfusion, The Second Affiliated Hospital of Harbin Medical University, Harbin, Heilongjiang 150086, China

**Keywords:** lipopolysaccharide, D-galactosamine, acute liver injury, catalpol, TNF-α

## Abstract

The purpose of this study was to investigate the protective effect of catalpol on Lipopolysaccharide (LPS)/D-galactosamine (D-gal)-induced acute liver injury in mice. The mouse model was established by injection of LPS and D-gal. Catalpol (2.5, 5, 10 mg/kg) were pretreated intraperitoneally 1 h before LPS and D-gal. The survival rate, AST, ALT, MDA, MPO activity, hepatic tissue histology, TNF-α level, and NF-κB activation were assayed. The results revealed that catalpol dose-dependently elevated the survival rate. Furthermore, catalpol reduced the activities of AST, ALT, MDA, and MPO. The production of TNF-α was also inhibited by treatment of catalpol. In addition, catalpol inhibited LPS/D-gal-induced NF-κB activation. The expression of Nrf2 and HO-1 were up-regulated by treatment of catalpol. These results indicated that pretreatment with catalpol could attenuate LPS/D-gal-induced acute liver injury in mice and the underlying mechanism may due to the inhibition of NF-κB signaling pathway and the activation of Nrf2 signaling pathway.

## INTRODUCTION

Fulminant hepatic failure was characterized by hepatic encephalopathy, coagulopathy and progressive multiorgan failure [1]. There are many causes of the disease, such as bacteria, viral hepatitis, alcohol and other hepatotoxic drugs. And the mortality of hepatic failure remains high. To conveniently study the pathogenesis of the disease, Lipopolysaccharide (LPS) in combination with D-galactosamine (D-gal) was used to induce an experimental liver injury in mice. Studies have shown that this model is similar to clinically fulminant hepatic failure [2, 3]. LPS, the important composition of endotoxin of Gram-negative bacteria, causes liver injury, and D-gal results in lipid peroxidation though triggering oxidative stress of hepatocytes [4, 5]. Based on the pathogenesis we know, specific and effective therapies are urgently needed to control this disease.

Catalpol, an effective active ingredient extracted from *Rehmannia glutinosa*, has been known to have multiple pharmacological activities, such as anti-inflammatory and anti-oxidative effects [6, 7]. Catalpol has been reported to protect against LPS-induced acute lung injury in mice [8]. Catalpol has been known to prevent D-galactose-induced mitochondrial dysfunction in mice [9]. Furthermore, catalpol was found to attenuate LPS-elicited rat microcirculation disorder by inhibiting inflammatory response [10]. In addition, a previous study showed that catalpol could attenuate ovalbumin-induced asthma in mice [11]. But few papers can be found about the effects of catalpol on fulminant hepatic failure induced by LPS and D-gal. Therefore, we aimed to investigate the potential role of catalpol on LPS and D-gal induced fulminant hepatic failure and to further illuminate its underlying mechanism.

## RESULTS

### Catalpol reduces the survival rate and attenuates hepatotoxicity against LPS/D-gal induced hepatic injury in mice

**Figure 1:**
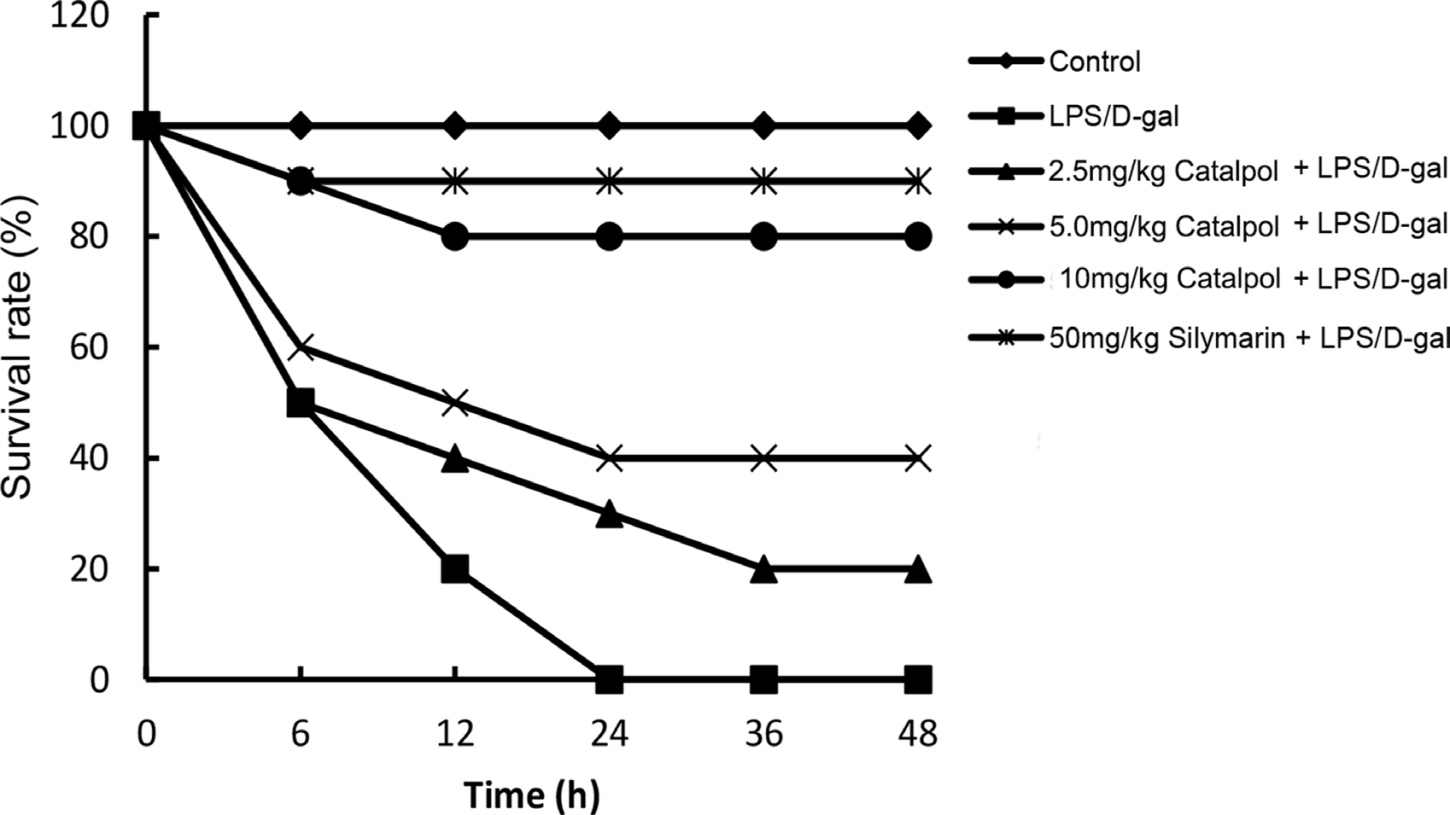
Effects of catalpol on LPS/D-gal induced mortality in mice (*n* = 12) Balb/c mice were pretreated intraperitoneally with catalpol (2.5, 5, 10 mg/kg) or PBS injection 1 h before LPS/D-gal administration.

**Figure 2:**
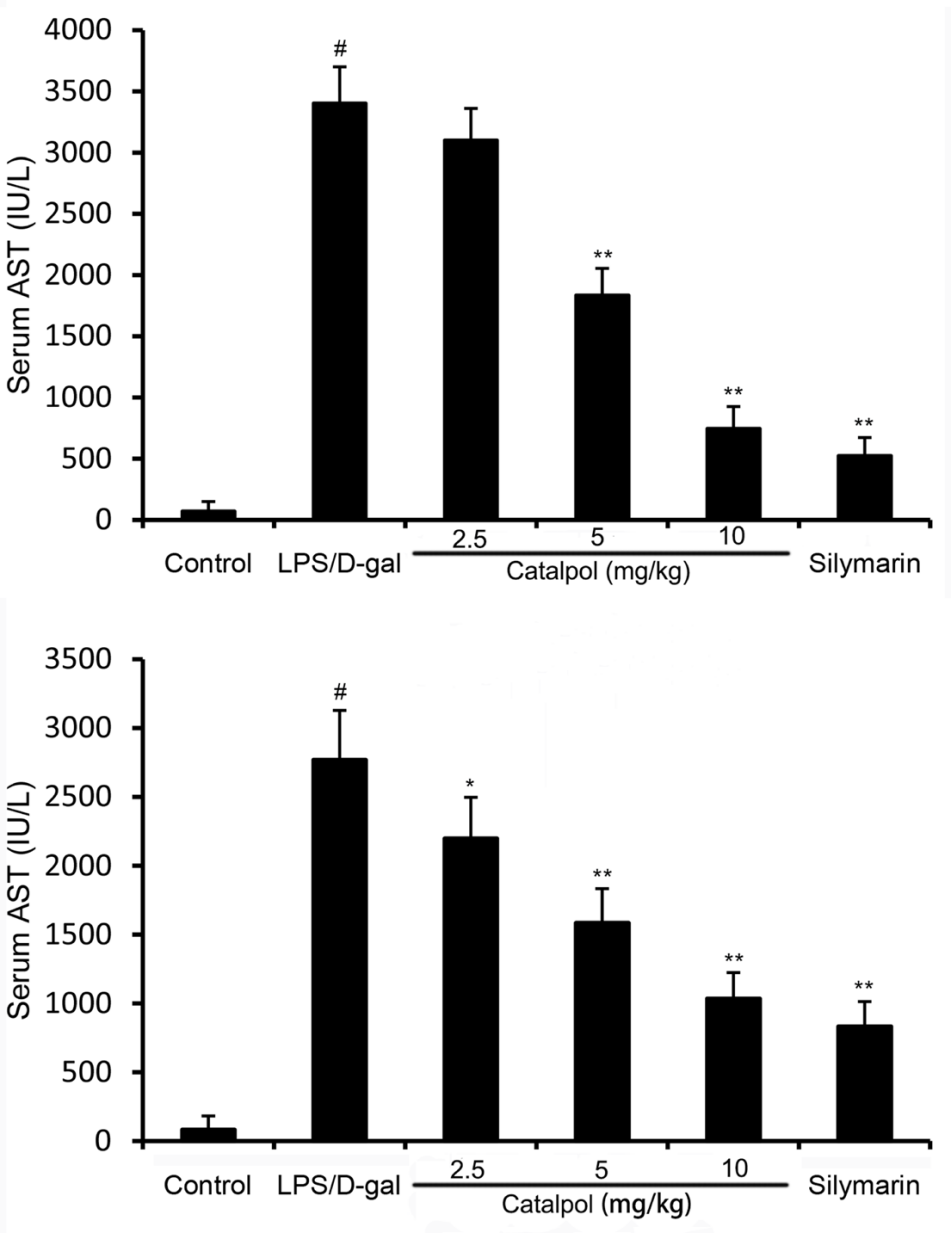
Effects of catalpol on ALT and AST activities in mice after LPS/D-gal treatment Balb/c mice were pretreated intraperitoneally with catalpol (2.5, 5, 10 mg/kg) or PBS injection 1 h before LPS/D-gal administration. The values presented are the mean ± SEM. *p*^#^ < 0.01 vs. control group, p^*^ < 0.05, p^**^ < 0.01 vs. LPS/D-gal group.

Animals challenged by LPS/D-gal all died at 24 h, which was improved by pretreatment of catalpol in a dose dependent manner (Figure 1). Serum ALT and AST activities had a significant increase at 6 h after LPS/D-gal administration. However, the increase was prevented by pretreatment of catalpol (Figure 2).

### Catalpol ameliorates the changes of hepatic tissue challenged by LPS/D-gal in mice

**Figure 3:**
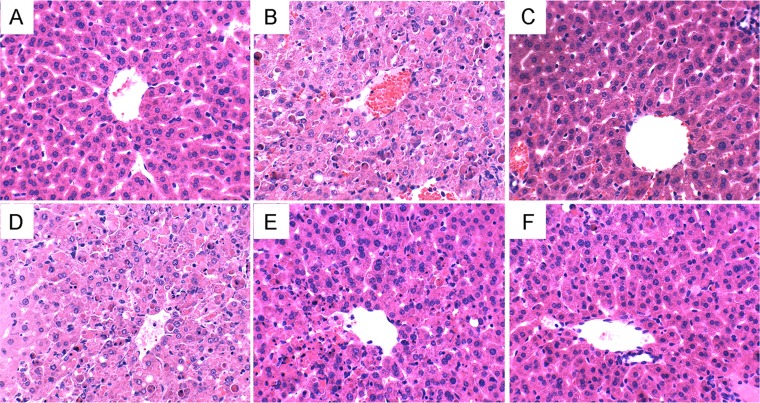
Histopathologic sections of thelivers (H&E, ×400) (**A**) Control group treated with PBS. (**B**) Group treated with LPS/D-gal. (**C**) Group treated with 50 mg/kg silymarin. (**D**) Group pretreated with 2.5 mg/kg catalpol 1 h before LPS/D-gal administration. (**E**) Group pretreated with 5 mg/kg catalpol 1 h before LPS/D-gal administration. (**F**) Group pretreated with 10 mg/kg catalpol 1 h before LPS/D-gal administration.

LPS/D-gal challenge caused a series of pathologic changes of liver, such as severe hemorrhagic necrosis, destruction of hepatic architecture, massive infiltration of inflammatory cells, while these changes were relatively slight with the pretreatment of catalpol (Figure 3).

### Catalpol decreases MDA production and MPO activity in LPS/D-gal induced mice

**Figure 4:**
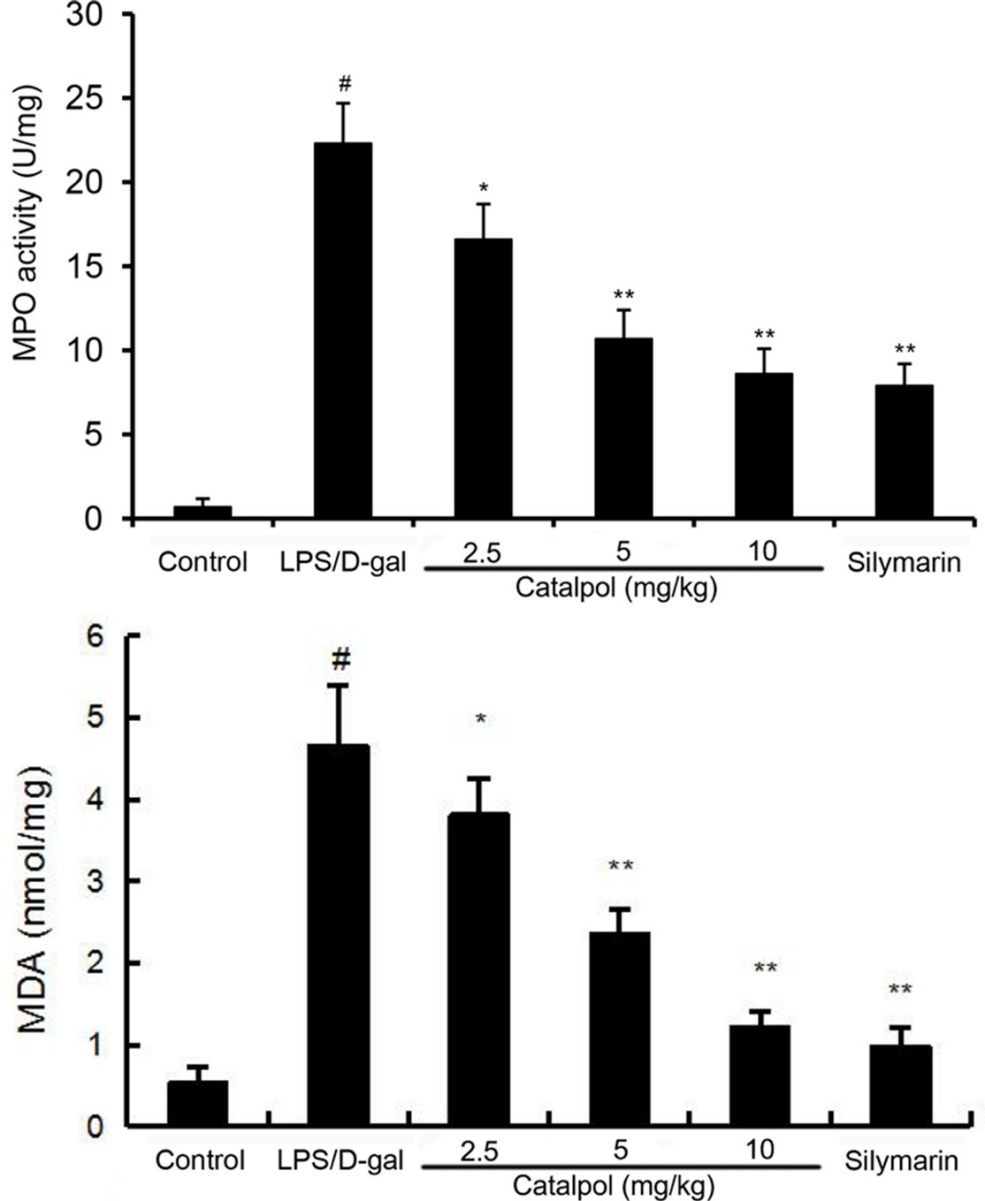
Effects of catalpol on MDA level and MPO activity in LPS/D-gal induced mice Mice were given an intraperitoneal injection of catalpol (2.5, 5, 10 mg/kg) or PBS 1 h before LPS administrationrespectively. The values presented are the mean ± SEM. *p*^#^ < 0.01 vs. control group, p^*^ < 0.05, p^**^ < 0.01 vs. LPS group.

It is known that MPO is a marker of tissue neutrophil infiltration. As shown in Figure 4, a significant increase of MPO activity was observed in mice after LPS/D-gal challenge compared with mice of the control group, whereas pretreatment with catalpol dose-dependently decreased MPO activity induced by LPS/D-gal. The effects of catalpol on MDA production were measured. As shown in Figure 4, a significant increase of MDA level was observed in mice after LPS/D-gal challenge compared with mice of the control group, whereas pretreatment with catalpol dose-dependently decreased MDA level induced by LPS/D-gal.

### Catalpol reduces TNF-α production induced by LPS/D-gal in mice

**Figure 5:**
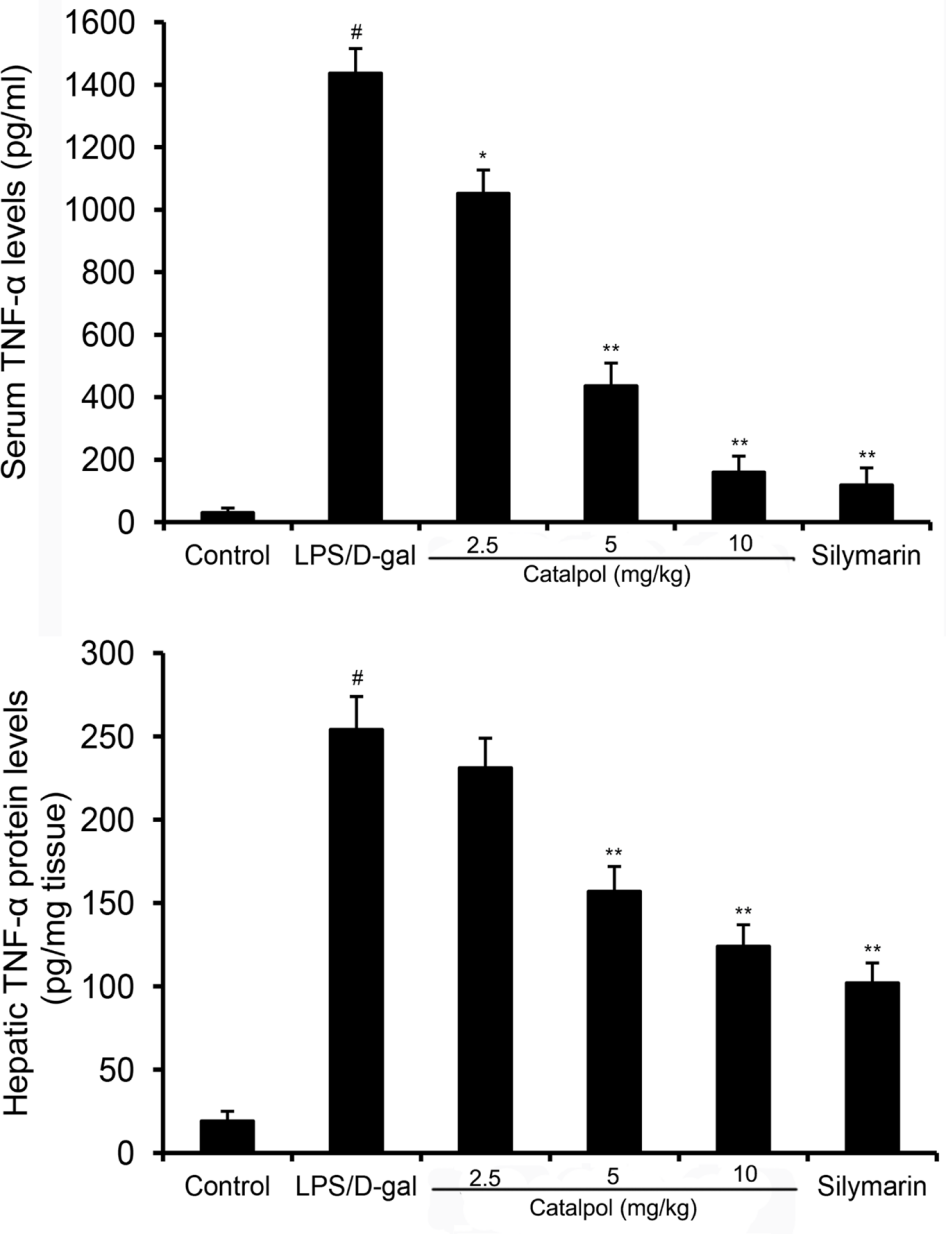
Effects of catalpol on serum and hepatic TNF-α levels in LPS/D-gal induced mice Mice were given an intraperitoneal injection of catalpol (2.5, 5, 10 mg/kg) or PBS 1 h before LPS administration respectively. The values presented are the mean ± SEM. *p*^#^ < 0.01 vs. control group, p^*^ < 0.05, p^**^ < 0.01 vs. LPS group.

TNF-α is a pivotal mediator in LPS/D-gal induced liver injury. In the present study, LPS/D-gal challenge significantly increased the levels of serum and hepatic TNF-α in mice. However, a decrease was observed in mice treated with catalpol in a dose-dependent manner (Figure 5).

### Catalpol inhibits NF-κB activation in LPS/D-gal induced mice

**Figure 6:**
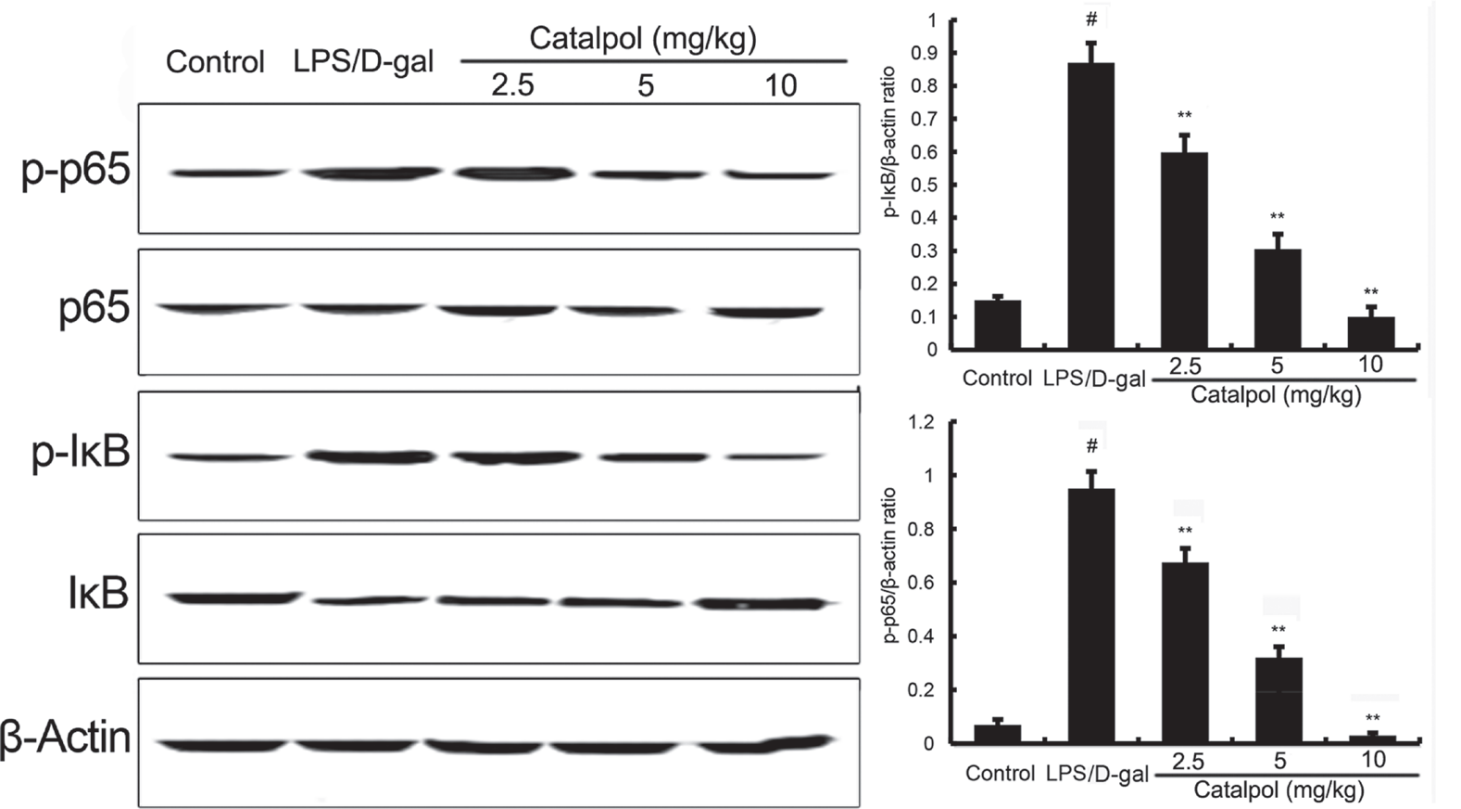
Effects of catalpol on LPS/D-gal induced NF-κB activation in mice Balb/c mice were pretreated intraperitoneally with catalpol (2.5, 5, 10 mg/kg) or PBS injection 1 h before LPS/D-gal administration. β-Actin was used as a control. The values presented are the mean ± SEM. *p*^#^ < 0.01 vs. control group, p^*^ < 0.05, p^**^ < 0.01 vs. LPS group.

NF-κB plays a key role in LPS/D-gal induced liver injury, which is a crucial transcriptional factor in the regulation of TNF-α transcription [12]. After LPS/D-gal challenge, phospho-NF-κB p65 and phospho-IκBα proteins were markedly increased in mice compared to that in the control group. Pretreatment with catalpol dose-dependently decreased the phosphorylation of NF-κB p65 and IκBα (Figure 6).

### Effects of catalpol on Nrf2 and HO-1 expression in LPS/D-gal induced mice

**Figure 7:**
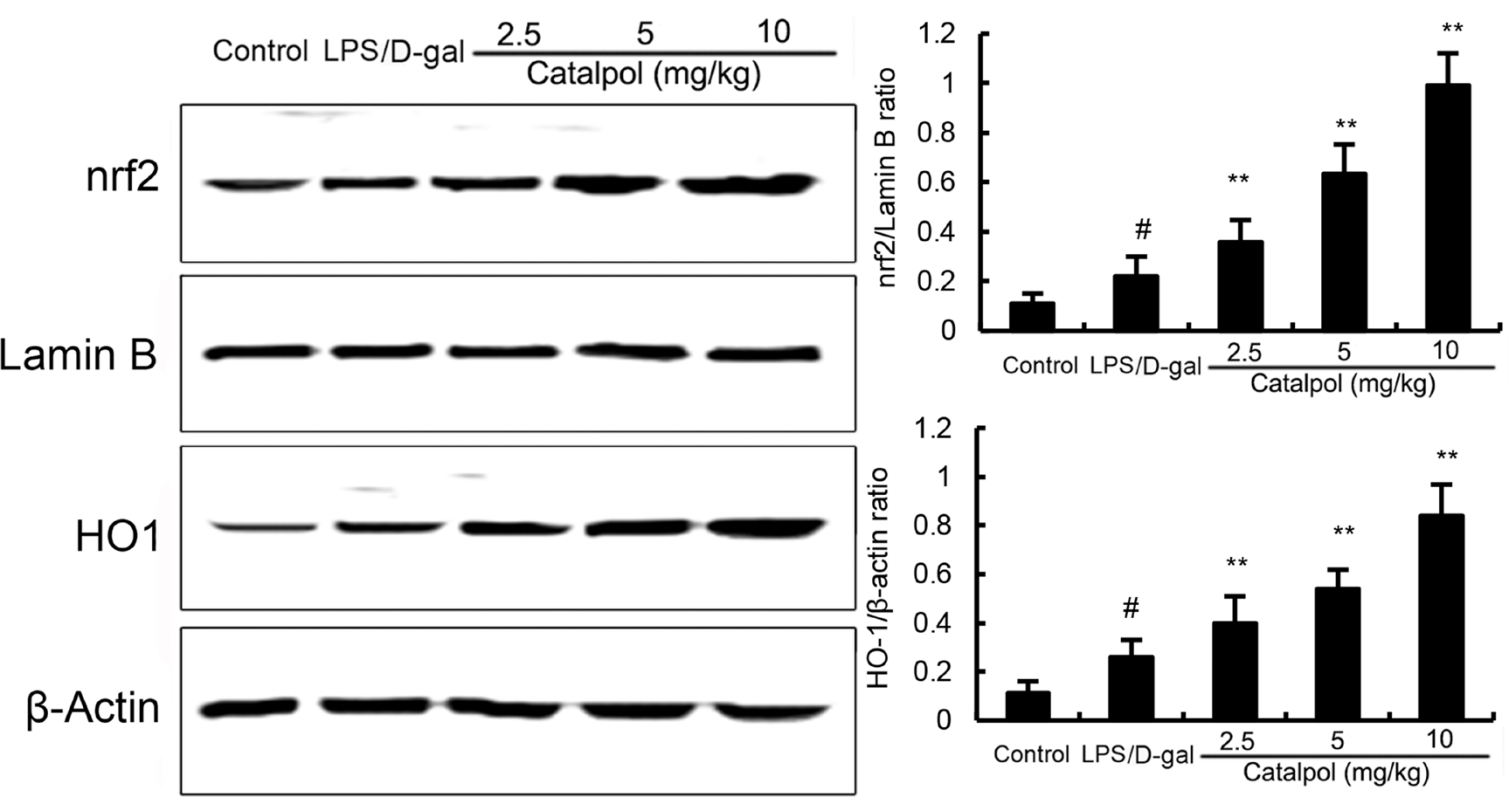
Effects of catalpol on Nrf2 and HO-1 expresssion in mice Balb/c mice were pretreated intraperitoneally with catalpol (2.5, 5, 10 mg/kg) or PBS injection 1 h before LPS/D-gal administration. β-Actin was used as a control. The values presented are the mean ± SEM. *p*^#^ < 0.01 vs. control group, p^*^ < 0.05, p^**^ < 0.01 vs. LPS group.

Nrf2 signaling pathway plays an important role in the regulation of oxidative response. In the present study, we detected the effects of catalpol on Nrf2 signaling pathway to assess the anti-oxidative mechanism of catalpol. After LPS/D-gal challenge, the expression of Nrf2 and HO-1 proteins were increased in mice compared to that in the control group. Pretreatment with catalpol dose-dependently increased the expression of Nrf2 and HO-1 (Figure 7).

## DISCUSSION

Galactosamine is a hexosamine derived from galactose with the molecular formula C_6_H_13_NO_5_. D-GalN is a specific hepatotoxic agent metabolized exclusively in hepatocytes, which reduces intracellular pool of uracil nucleotides, thus inhibiting the synthesis of RNA and proteins. LPS, a major constituent of *Gram-negative* bacterial membrane, specifically activates Toll-like receptor 4, leading to the production of cytokines which in turn regulate inflammatory and innate and subsequent adaptive immune responses. It is reported that LPS in combination with D-gal highly sensitizes animals to develop lethal liver injury showing biochemical and metabolic changes similar to fulminant hepatic failure [13]. In the present study, we aimed to investigate the effects of catalpol on fulminant hepatic failure based on this animal model induced by LPS/D-gal. Pretreatment with catalpol attenuated LPS/D-gal induced liver injury, as indicated by the reduction of lethality, ALT/AST activities, MPO activity and tissue pathological injury.

TNF-α is known to be a pleiotropic cytokine that contributes to the triggering of an inflammatory cascade involving the induction of cytokines including IL-1, IL-6, IFN-γ, nitric oxide and cell adhesion molecules [14]. At the later stages of liver injury, TNF-α-induces neutrophil transmigration, which plays a crucial role in hepatocyte necrosis [15]. Furthermore, the key role of TNF-α in LPS/D-gal induced hepatotoxicity has been confirmed by experiments with TNF-α knockout or TNF-receptor p55 knockout mice [16, 17]. Our results showed that serum and hepatic TNF-α levels had a markedly increase in LPS/D-gal induced mice. However, pretreatment with catalpol dose-dependently decreased the levels. Therefore, catalpol may protect liver injury through inhibiting TNF-α production in LPS/D-gal induced mice. MDA, a biomarker of oxidative stress, was measured to assess the anti-oxidative effects of catalpol. The results suggested that catalpol inhibited LPS/D-gal-induced oxidative response in mice. These results indicated that catalpol protected against LPS/D-gal-induced fulminant hepatic failure by suppressing inflammatory and oxidative responses.

NF-κB is implicated in the regulation of inflammatory and immune responses which plays an important role in serious diseases [18, 19]. In resting cells, NF-κB family members are sequestered in the cytoplasm, for they are bound to their inhibitors which belong to the IκB family. Upon cell activation, IκB proteins are phosphorylated and degraded by the proteasome. Subsequently, NF-κB could enter the nucleus and bind to the DNA, resulting in promoting transcription [20]. It is known that TNF-α transcription stimulated by LPS is regulated by NF-κB [21]. Moreover, studies have shown that NF-κB played a vital role in LPS/D-gal liver injury [22, 23]. To further explore the mechanism of catalpol on liver injury in LPS/D-gal induced mice, NF-κB was assayed by western blot. The results revealed that NF-κB activation was dose-dependently inhibited by pretreatment with catalpol in LPS/D-gal induced mice. Hence, we speculated that the reduction of TNF-α production by catalpol may be associated with the inhibition of NF-κB activation. Nrf2 signaling pathway was involved in the development of oxidative response [24]. Activation of Nrf2 has been reported to protect against liver injury [25, 26]. In the present study, our results showed that catalpol could increase the expression of Nrf2 and HO-1. The results suggested that catalpol inhibited oxidative stress by activating Nrf2 signaling pathway.

In conclusion, the protective effect of catalpol against LPS/D-gal induced liver injury in mice appeared to be closely related to the inhibition of NF-κB activation and activation of Nrf2. To acquire a better understanding of the effect of catalpol on LPS/D-gal induced liver injury, further and more work will be required.

## MATERIALS AND METHODS

### Reagents

Catalpol (purity ≥ 98%) was purchased from Sigma (St. Louis, MO, USA) and prepared with dimethyl sulfoxide (DMSO). LPS (Escherichia coli, 0111:B4) and D-gal were obtained from Sigma (St. Louis, MO, USA). Alanine aminotransferase (ALT), aspartate aminotransferase (AST) assay kits were purchased from Nanjing Jiancheng Bioengineering Institute (Nanjing, China). The myeloperoxidase (MPO) kit was provided by the Jiancheng Bioengineering Institute of Nanjing (Nanjing, Jiangsu, China). Mouse TNF-α enzyme-linked immunosorbent assay (ELISA) kit was purchased from BioLegend (CA, USA). Rabbit anti-mouse phospho-NF-κB p65, anti-mouse IκBα, mouse mAbphospho-IκBα, anti-mouse Nrf2, anti-mouse HO-1, and rabbit anti-mouse beta-actin antibody were purchased from Cell Signaling Technology Inc. (Beverly, MA). HRP-conjugated goat anti-rabbit and goat-mouse antibodies were provided by GE Healthcare (Buckinghamshire, UK).

### Animals

Specific pathogen-free male BALB/c mice (6-8 weeks old; 18–20 g) were purchased from the Center of Experimental Animals of Harbin Medical University (Harbin, China). The mice were kept in controlled conditions (23°C, 55% humidity and 12 h day/night cycle). Prior to experiments, all mice were accustomed to the new environment for at least 1 week, and supplied with standard rodent feed and tap water ad libitum. The animal study protocol was approved by the Harbin Medical University Animal Care and Use Committee and all animal experiments were performed in accordance with the National Institutes of Health guide for the Care and Use of Laboratory Animals.

### Experimental design

Seventy-two mice were divided into six groups and each group contained twelve mice. Mice were administered with catalpol (2.5, 5, 10 mg/kg) dissolved in 250 μl phosphate-buffered saline (PBS) once daily for three days prior to challenge experimentation. The dose of catalpol used in this study was based on our preliminary experiment and previous study [8]. The mice of control group were given an equal volume of PBS. Mice given an isometric silymarin (50 mg/kg) were used as positive control. Fulminant hepatic failure in mice were induced by the intraperitoneal injection of LPS (50 μg/kg) and D-gal (800 mg/kg) combination dissolved in PBS. The survival rate of the mice was evaluated within 48 h after LPS/D-gal injection.

### Analysis of liver enzymes

Blood was collected at 6 h after LPS/D-gal injection. Serum ALT and AST levels were measured by using corresponding assay kits according to the manufacturer’s instructions.

### Histological evaluation

Liver tissue was collected at 6 h after LPS/D-gal injection and fixed in 10% neutral-buffered formalin. Then the samples were sliced into 5 μm sections and stained with hematoxylin–eosin. Finally, the pathologic changes were observed under a light microscope.

### MPO assay

Liver samples were homogenized in PBS and centrifuged at 10000 × g for 10 min. The supernatant was collected for further determinations and the resultant pellets were removed. MPO activity in liver tissues was assayed following the manufacturer’s protocol of MPO kit.

### Measurement of TNF-α level

Serum and hepatic tissues were collected for the detection of TNF-α. The TNF-α level in serum and hepatic tissues was measured by mouse TNF-α ELISA kit according to the manufacturer’s protocol.

### Western blotting

The liver tissue was homogenated with a lysis buffer, and then centrifuged at 3000 × g for 10 min at 4°C. The supernatant was collected and the total protein concentration was measured using a BCA protein assay kit. For western blotting, the detail procedures were similar to previous study [27]. The protein levels of Nrf2, HO-1, p65, phosphorylated-p65, IκBα, phosphorylated-IκBα were assayed by western blotting.

### Statistical analysis

Data were expressed as mean ± SEM. Differences between the mean values of normally distributed data were assessed by one-way ANOVA (Dunnett’s *t* test) and the two-tailed Student’s *t* test. Statistical significance was set at *p* < 0.05.
